# Space evaluation in football games via field weighting based on tracking data

**DOI:** 10.1038/s41598-021-84939-7

**Published:** 2021-03-09

**Authors:** Takuma Narizuka, Yoshihiro Yamazaki, Kenta Takizawa

**Affiliations:** 1 Department of Physics, Faculty of Science and Engineering, Chuo University, Bunkyo, Tokyo 112-8551 Japan; 2 Department of Physics, School of Advanced Science and Engineering, Waseda University, Shinjuku, Tokyo 169-8555 Japan

**Keywords:** Statistical physics, Statistics

## Abstract

In football game analysis, space evaluation is an important issue because it is directly related to the quality of ball passing or player formations. Previous studies have primarily focused on a field division approach wherein a field is divided into dominant regions in which a certain player can arrive prior to any other players. However, the field division approach is oversimplified because all locations within a region are regarded as uniform herein. The objective of the current study is to propose a fundamental framework for space evaluation based on field weighting. In particular, we employed the motion model and calculated a minimum arrival time $\tau$ for each player to all locations on the football field. Our main contribution is that two variables $\tau _{\text{of}}$ and $\tau _{\text{df}}$ corresponding to the minimum arrival time for offense and defense teams are considered; using $\tau _{\text{of}}$ and $\tau _{\text{df}}$, new orthogonal variables $z_{1}$ and $z_{2}$ are defined. In particular, based on real datasets comprising of data from 45 football games of the J1 League in 2018, we provide a detailed characterization of $z_{1}$ and $z_{2}$ in terms of ball passing. By using our method, we found that $z_{1}(\vec {x}, t)$ and $z_{2}(\vec {x}, t)$ represent the degree of safety for a pass made to $\vec {x}$ at *t* and degree of sparsity of $\vec {x}$ at *t*, respectively; the success probability of passes could be well-fitted using a sigmoid function. Moreover, a new type of field division approach and evaluation of ball passing just before shots using real game data are discussed.

## Introduction

The game of football, which is also commonly referred to as soccer, is a complex system wherein 22 players in two different teams interact with each other in order for their team to win. In the process of carrying a ball to their respective goal, a vast number of movement options are available to players, leading to the emergence of various behaviors from individual to team levels, such as ball passing, dribbling, marking an opponent player, and organizing into formations. The emergence of such behaviors in football, and recent developments in data acquisition methodologies and tools^1^ have promoted a wide range of analyses for football games from the perspective of statistics as well as physics^2,3^. Examples of such analyses include the universality of goal distribution^4^, complex network analysis for ball passing^5,6^, dynamics-based analysis of ball motion^7,8^, and characterization of formations^9,10^ . (We refer to a term, “formation”, as the relative positioning of players expressed in a form such as 4–4–3).

In football games, the difficulty of receiving a pass or making a shot depends on the players’ formations or the position of the ball. It is natural to assume that each location on the field has some value related to the game condition. Thus characterization of space on the field by such a value is a key issue in football game analysis. However, the definition of space in the context of football is not well-defined and neither are approaches for its evaluation. In general, there are two approaches for space evaluation, namely field division and field weighting. A well-known approach for field division is determining a “dominant region”—as introduced by Taki et al.^11,12^—wherein a certain player can arrive in a region prior to any other players. A typical example of field division is defining a Voronoi region for each player, which corresponds to their dominant region as defined by the Euclidean distance between their position and each location in the field^13^. The Voronoi region provides a first approximation of the territory of any player on the field. Previous studies have investigated its basic properties in football games; for example, Kim have characterized the area and number of vertices of Voronoi regions^14^ and Fonseca et al. have performed a time series analysis for Voronoi regions^15^.

However, because these Voronoi regions lack information about the velocity and acceleration of players, Fujimura and Sugihara proposed a more realistic definition of the dominant region based on a “motion model”^16^. In the motion model, each player is assumed to move according to an equation of motion with acceleration and resistance terms. Thus, given the initial position and velocity of a player, their arrival times at any location on the field can be calculated based on the equation of motion. Accordingly, using the equations of motion, the Voronoi regions of players can be suitably modified as per their velocity and acceleration. Using the motion model, Fujimura and Sugihara have investigated the receivable pass variation which quantifies the number of passes a player can receive^16^ and Ueda et al. have examined the relationship between the dominant region and offensive performance of a team^17^. Another approach for extension of the definition of a dominant region is a data-driven one wherein individual dominant regions on the field could be estimated using machine learning^18,19^.

Though field division based on dominant regions is one approach for space evaluation when positions and motions of players are known, it is an oversimplified approach, because all locations within a dominant region yield the same space evaluation in that only one specific player can reach a location first. For further detailed characterization of space, the field division approach could be replaced by a field weighting approach using appropriate variables. Several recent studies have addressed the problem of field weighting based on variables such as pass probability^20^, scoring opportunity^21^, and space occupation and generation gain^22^. However, a field weighting using arrival times of players could extend the field division approach in a more straightforward way.

In this study, we proposed a framework for space evaluation based on field weighting, which provides a definition of space from a new perspective. In particular, our space evaluation approach is based on a “minimum arrival time” $\tau$ to all locations in the field, which can be calculated based on the physics-based motion model. Herein, we considered two variables $\tau _{\text{of}}$ and $\tau _{\text{df}}$ corresponding to the minimum arrival time for offense and defense teams, respectively. Using $\tau _{\text{of}}$ and $\tau _{\text{df}}$, we introduced new orthogonal variables $z_{1}$ and $z_{2}$ that represent degrees of safety and sparsity of each location, respectively. To elucidate the quantitative meaning of $z_{1}$ and $z_{2}$, we carried out ball-passing analyses based on real datasets. As applications of our proposed space evaluation method, a new field division approach is discussed and an evaluation of ball passing just before shots for a dataset of football games is presented.

## Methods

### Dataset and system

The following characterization and demonstration of our space evaluation framework was based on datasets comprising data from 45 football games of the top division of the J League in 2018; these datasets were provided by DataStadium Inc., Japan^23^. This top division consists of 18 teams, then 9 games are performed in one section. We had the dataset of five sections, therefore 45 games, which were held between Aug. 10, 2018 and Sept. 2, 2018. We note that J League stands for the Japan Professional Football League, and it has now matured enough to attract top players from all over the world. Each dataset contains information about the absolute positional coordinates (*x*, *y*) of all players every 0.04 s as well as play-by-play data; the spatial resolution of the data is on the centimeter scale. DataStadium Inc. was authorized to collect and sell this data under a contract with the J League. This contract also ensures that the use of relevant datasets does not infringe on any rights of the players and clubs belonging to the J League. Though the datasets are proprietary, we received explicit permission from DataStadium Inc. for their use in this research. The data analyses and visualizations in this study were performed using Python packages on a MacBook Pro system with a 2-GHz Intel Core i5 processor and 16 GB of memory.

### Motion model

Here, we summarize the motion model proposed by Fujimura and Sugihara, which is also used in this study^16^. In this motion model, each player is assumed to move according to the following equation of motion:

1 $$\begin{aligned} m \frac{d^{2} \vec {x}(t)}{d t^{2}}&= F \vec {n} - k \frac{d \vec {x}(t)}{d t} \end{aligned}$$

where *m* is the mass of the player and $\vec {x}(t)$ is the position of the player at time *t*; *F* and $\vec {n}$ are the magnitude and direction of the attractive force, respectively; and *k* is the coefficient for viscous resistance. Namely, we assume that the player accelerates in the direction of $\vec {n}$ with magnitude *F* and becomes harder to accelerate in proportion to its velocity $d\vec {x}(t)/dt$. The solution for Eq. (1) for an initial position $\vec {x}_{0}$ and initial velocity $\vec {v}_{0}$ is expressed as follows:

2 $$\begin{aligned} \vec {x}(t) = \vec {x}_{0} + \frac{1 - \exp (-\alpha t)}{\alpha } \vec {v}_{0} + V_{\text{max}} \left( t - \frac{1 - \exp (-\alpha t)}{\alpha }\right) \vec {n}, \end{aligned}$$

where $V_{\text{max}}=F/m$ and $\alpha = k/m$ are arbitrary constants that correspond to the player’s terminal velocity and the inverse of the relaxation time to $V_{\text{max}}$, respectively. Thus, given $V_{\text{max}}$ and $\alpha$, we can obtain arrival times of each player to all locations using Eq. (2). In the study by Fujimura and Sugihara, they set $V_{\text{max}}=7.8$ [m/s] and $\alpha =1.3$ [1/s]; these values have been used for typical values during sprinting by the players and do not vary significantly among players^16^.

## Results

### Definition of variables

Let $\tau _{a}(\vec {x}, t)$ be the minimum arrival time for a player *a* to a location $\vec {x}$ at time *t*. Accordingly, $\tau _{a}(\vec {x}, t)$ is defined as the minimum time required for the player *a* to move from their position at *t* to $\vec {x}$. In order to calculate $\tau _{a}(\vec {x}, t)$, we employed the solution of the aforementioned motion model (i.e., Eq. 2). For simplicity, we set $V_{\text{max}}=7.8$ [m/s] and $\alpha =1.3$ [1/s] for all players by assuming that they move to all locations by sprinting. This simplification is considered appropriate for grasping the essential features of our space evaluation framework (i.e., the characterization of the meanings of $z_{1}$ and $z_{2}$) because $V_{\text{max}}$ and $\alpha$ do not vary significantly among players. However, for a more practical application of our framework such as assessing the playing ability of players, the use of more realistic values for $V_{\text{max}}$ and $\alpha$ based on the play in different games is recommended.

**Figure 1:**
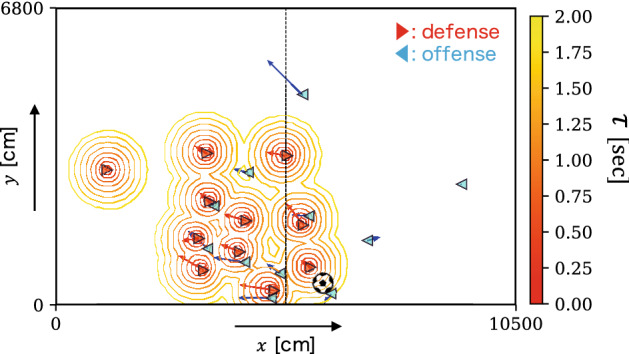
Visualization of $\tau _{\text{df}}$ at a certain time as a contour plot over a range of 2 s. Players in offense (i.e., ball possession team) and defense teams are depicted using blue leftward and red rightward triangles, respectively. In this situation, the player on the right side near the halfway line has the ball and is trying to pass it to the other side.

Furthermore, we also define the minimum arrival time for a team *A* to $\vec {x}$ at time *t* as $\tau _{A}(\vec {x}, t) \equiv \min _{a \in A} \tau _{a}(\vec {x}, t)$. In particular, we denote the minimum arrival time for the defense and offense teams as $\tau _{\text{df}}(\vec {x}, t)$ and $\tau _{\text{of}}(\vec {x}, t)$, respectively; here, the offense team is defined as the team that has possession of the ball. Figure 1 depicts a visualization for $\tau _{\text{df}}$ at a certain time as a contour plot over a range of 2 s.

**Figure 2:**
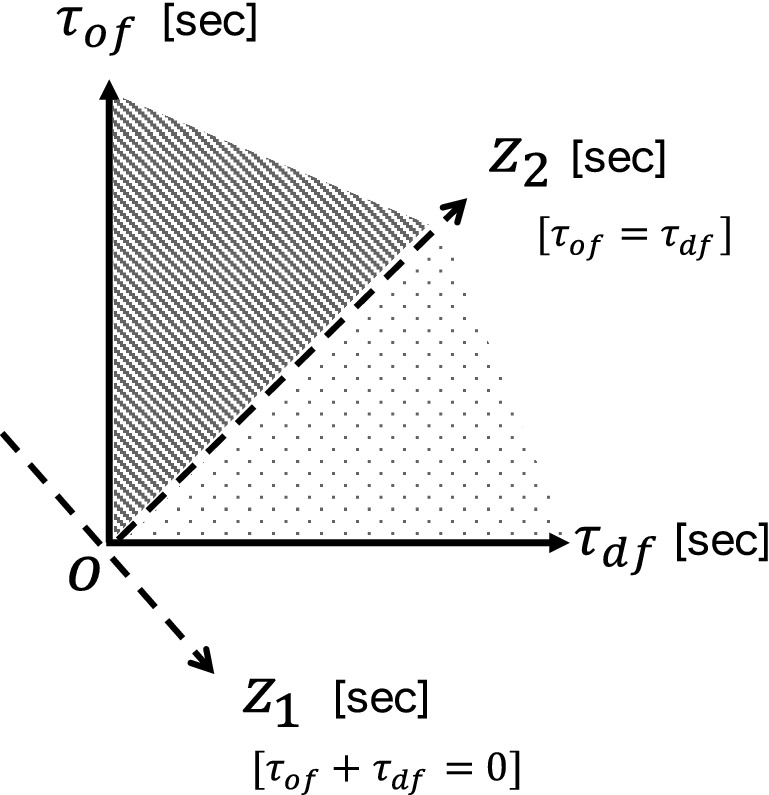
Relationship between the axes of $$ \tau _{\text{of}}$$ and $$\tau _{\text{df}} $$ and those of $$ z_{1}$$ and $$z_{2}$$. The safe space ($$ z_{1} > 0 $$) and the risky space ($$ z_{1} < 0 $$) for the offense team are shown by shading.

Now, we evaluate the location $\vec {x}$ at time *t* using the two variables $\tau _{\text{df}}(\vec {x}, t)$ and $\tau _{\text{of}}(\vec {x}, t)$. In Fig. 2, the domain $\tau _{\text{of}}(\vec {x}, t) < \tau _{\text{df}}(\vec {x}, t)$ corresponds to the “safe space” for the offense team in that an offense player can arrive in this domain prior to any defense player. Thus, the degree of safety of $\vec {x}$ at *t* for the offense team can be quantified via a signed distance $z_{1}(\vec {x}, t)$ from the axis $\tau _{\text{of}}(\vec {x}, t) = \tau _{\text{df}}(\vec {x}, t)$ as follows:

3 $$\begin{aligned} z_{1}(\vec {x}, t) = \frac{\tau _{\text{df}}(\vec {x}, t) - \tau _{\text{of}}(\vec {x}, t)}{\sqrt{2}}. \end{aligned}$$

When $z_{1} > 0$, $\tau _{\text{of}} < \tau _{\text{df}}$. Therefore, an offense player can reach faster than a defense player at the space satisfying $z_{1} > 0$. That is, the space for $z_{1} > 0$ is safe for the offense player. In Fig. 2, the safe space ($z_{1} > 0$) and the risky space ($z_{1} < 0$) for the offense team are shown by shading. Similarly, another variable $z_{2}(\vec {x}, t)$, which is orthogonal to $z_{1}(\vec {x}, t)$, can be defined as follows:

4 $$\begin{aligned} z_{2}(\vec {x}, t) = \frac{\tau _{\text{df}}(\vec {x}, t) + \tau _{\text{of}}(\vec {x}, t)}{\sqrt{2}}. \end{aligned}$$

Because $z_{2}(\vec {x}, t)$ is proportional to $\tau _{\text{df}} + \tau _{\text{of}}$, the larger $z_{2}(\vec {x}, t)$ is, the more time it takes for the players to reach $\vec {x}$. Therefore, $z_{2}(\vec {x}, t)$ roughly quantifies the degree of sparsity of the location $\vec {x}$ at *t*. The relationship between the axes of $\tau _{\text{of}}$ and $\tau _{\text{df}}$ and those of $z_{1}$ and $z_{2}$ is depicted in Fig. 2. It is noteworthy that the definitions of $z_{1}$ and $z_{2}$ are independent of the definition of the motion model even though we employ the motion model proposed by Fujimura and Sugihara for one specific case in this study.

### Calculation of $z_{1}$ and $z_{2}$ using ball-passing data

**Figure 3:**
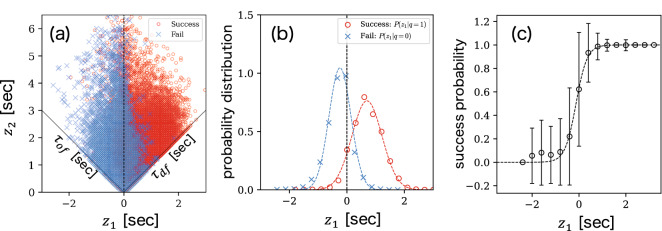
(**a**) Scatter plot of $z_{1}(\vec {x}_{\text{e}}, t_{\text{o}})$ and $z_{2}(\vec {x}_{\text{e}}, t_{\text{o}})$ for 34,189 passes obtained from 45 football games. Plots for failed and successful passes almost distribute in $z_{1} < 0$ and $z_{1} > 0$ domains, respectively. (**b**) Probability distributions of $z_{1}(\vec {x}_{\text{e}}, t_{\text{o}})$ for successful and failed passes. The dotted curves are the fitted normal distribution curves. (**c**) Success probability of passes as a function of $z_{1}(\vec {x}_{\text{e}}, t_{\text{o}})$. The success probabilities are calculated by averaging the value of *q* over each $z_{1}$. The dotted curve is the fitted sigmoid function given by Eq. (5), indicating that $z_{1}(\vec {x}, t)$ signifies the degree of safety for a pass made to $\vec {x}$ at time *t*.

For further characterization of $$ z_{1} $$ and $$z_{2} $$ using real-world ball-passing data, we created time-series data using 34,189 ball passes made in 45 football games that form part of the datasets used in our study. A row in the datasets corresponding to a pass is expressed as $$ [t_{\text{o}}, \vec {x}_{\text{o}}, t_{\text{e}}, \vec {x}_{\text{e}}, q] $$. Here, $$ t_{\text{o}} $$ and $$ \vec {x}_{\text{o}} $$ represent the time and positional coordinates for the origin of the pass, while $$ t_{\text{e}} $$ and $$ \vec {x}_{\text{e}} $$ represent those for the end of the pass, respectively. The variable $$ q \in \{1, 0\} $$ indicates the success (1) or failure (0) of a pass. For each pass, we describe the state of the end of the pass ($$ \vec {x}_{\text{e}} $$) at the moment the pass is made ($$ t_{\text{o}} $$) using $$ z_{1}(\vec {x}_{\text{e}}, t_{\text{o}}) $$ and $$ z_{2}(\vec {x}_{\text{e}}, t_{\text{o}}) $$. In order to investigate the relationship between the state of the end position and outcome of the pass (success or failure), we calculated $$ z_{1}(\vec {x}_{\text{e}}, t_{\text{o}}) $$ and $$ z_{2}(\vec {x}_{\text{e}}, t_{\text{o}}) $$ for all successful and failed passes using Eqs. (3) and (4), respectively; Fig. 3a shows the results of these calculations as a scatter plot.

### Meaning of $z_{1}(\vec {x}_{\text{e}}, t_{\text{o}})$

Figure 3b presents the probability distributions of $z_{1}(\vec {x}_{\text{e}}, t_{\text{o}})$ for successful and failed passes, which are denoted by $P(z_{1}|q=1)$ and $P(z_{1}|q=0)$, respectively. From the figure, we can observe that both distributions can be fitted well using normal distribution functions. From the corresponding normal curves, the mean and standard deviation for successful passes are obtained as $\mu _{1}=0.69$ and $\sigma _{1}=0.54$, while those for failed passes are $\mu _{0}=-0.25$ and $\sigma _{0}=0.38$. It is noteworthy that the peak values of these normal curves—which correspond to the mean values—are located at $z_{1} > 0$ and $z_{1} < 0$ for successful and failed passes, respectively. The sign of $z_{1}(\vec {x}_{\text{e}}, t_{\text{o}})$ depends on whether a player of the offense or defense teams reaches the ball sooner. Thus, it is reasonable that the sign of $\mu$ is strongly correlated with the outcome of the pass. Furthermore, we estimated the success probability $P(q=1|z_{1})$ of passes as a function of $z_{1}(\vec {x}_{\text{e}}, t_{\text{o}})$ by averaging the value of *q* over each $z_{1}$. Figure 3c shows our estimation results for the success probability of passes; from these results, it was observed that these success probability values could be fitted well using a sigmoid function, which is given by:

5 $$\begin{aligned} P(q=1|z_{1}) = \frac{1}{1 + \text {exp}[-(az_{1}+b)]} \end{aligned}$$

where $a=4.68$ and $b=0.48$. Thus, with respect to ball passing, $z_{1}(\vec {x}, t)$ signifies the degree of safety for a pass made to $\vec {x}$ at time *t*. In addition, this result suggests that the outcome of a particular pass can be estimated via logistic regression using $z_{1}$.

### Meaning of $z_{2}(\vec {x}_{\text{e}}, t_{\text{o}})$

As mentioned earlier, $z_{2}(\vec {x}_{\text{e}}, t_{\text{o}})$ indicates the degree of sparsity of $\vec {x}_{\text{e}}$ at $t_{\text{o}}$. To elucidate the meaning of sparsity, we calculate the following quantity for each pass:

6 $$\begin{aligned} \tilde{R} = \frac{|| \vec {x}_{\text{e}} - \vec {x}_{c}(t_{\text{o}}) ||}{\sigma (t_{\text{o}})}. \end{aligned}$$

Here, $\vec {x}_{c}$ is the centroid position for all 20 field players excluding the goal keepers; $\sigma$ is the standard deviation from $\vec {x}_{c}$, which corresponds to the size of the formation; these variables are defined as follows:

7 $$\begin{aligned} \vec {x}_{c}(t)&= \frac{1}{N}\sum _{j=1}^{20} \vec {x}_{j}(t), \end{aligned}$$

8 $$\begin{aligned} \sigma (t)&= \sqrt{\frac{1}{20} \sum _{j=1}^{20}|\vec {x}_{c}(t) - \vec {x}_{j}(t)|^{2}}. \end{aligned}$$

**Figure 4:**
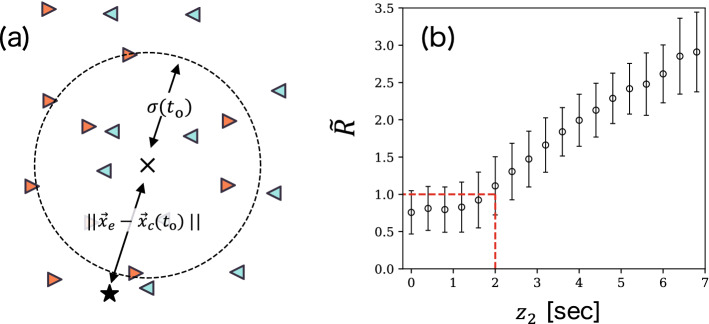
(**a**) Schematic representation of the definition of $\tilde{R}$ (Eq. 6). Players in two teams are represented by red rightward and blue leftward triangles. The cross and star markers represent the end points of $\vec {x}_{c}(t_{\text{o}})$ and $\vec {x}_{\text{e}}$, respectively. The dotted line indicates the circle with radius $\sigma (t_{\text{o}})$. This case corresponds to $\tilde{R} > 1$, meaning that the end point of the pass is located outside the formation. (**b**) Relationship between $\tilde{R}$ and $z_{2}(\vec {x}_{\text{e}}, t_{\text{o}})$. $z_{2}(\vec {x}, t) \simeq 2$ is the threshold that determines whether the location $\vec {x}$ at *t* is inside or outside the formation. We roughly regard the spaces with $z_{2} \gtrsim 2$ and $z_{2} \lesssim 2$ as sparse and dense, respectively.

Figure 4a shows the schematic representation of the definition of $\tilde{R}$. When $\tilde{R}$ is less (more) than unity, the end point $\vec {x}_{\text{e}}$ of the pass is roughly located inside (outside) the formation.

The relationship between $\tilde{R}$ and $z_{2}$ obtained based on our data is shown in Fig. 4b. It was observed that the value of $z_{2}$ at which $\tilde{R} \simeq 1$ is $\simeq 2$, i.e., $\tau _{\text{df}}+\tau _{\text{of}} \simeq 2\sqrt{2}$; thus, $z_{2}(\vec {x}, t) \simeq 2$ is the threshold that determines whether the location $\vec {x}$ at *t* is inside or outside the formation. Based on this result, we roughly regard the spaces with $z_{2} \gtrsim 2$ and $z_{2} \lesssim 2$ as sparse and dense spaces, respectively.

## Discussion

The objective of the current study was to propose a fundamental framework for space evaluation based on field weighting. The properties and significance of our proposed framework for space evaluation are described below. First, instead of a field division approach, our framework is based on a field weighting approach derived from a previous motion model. Second, the variables for field weighting have an explicit physical meaning, i.e., minimal arrival times of players when they move to all locations by sprinting. Third, each location is evaluated using two variables; $\tau _{\text{df}}$ and $\tau _{\text{of}}$, and the influence of both the defense and offense teams at each location can be simultaneously evaluated using our approach. Fourth, most importantly, two orthogonal variables $z_{1}$ and $z_{2}$, which correspond to the degrees of safety and sparsity of a location, were introduced. It is important to note that our framework does not depend on the definition of a motion model; in addition, $z_{1}$ and $z_{2}$ provide a quantitative definition of space on the football field. Therefore, our framework essentially yields a starting point to answer the question, “what is space in football games?”

**Figure 5:**
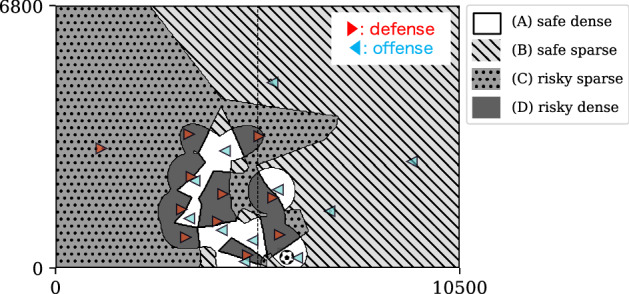
Typical example of field division into the following four spaces: (A) safe dense space ($z_{1} > 0$ and $z_{2} < 2$), (B) safe sparse space ($z_{1} > 0$ and $z_{2} > 2$), (C) risky sparse space ($z_{1} < 0$ and $z_{2} > 2$), and (D) risky dense space ($z_{1} < 0$ and $z_{2} < 2$). The players in the offense (i.e., ball-possession team) and defense teams are shown via blue leftward and red rightward triangles, respectively. The player on the right side near the halfway line has the ball. In order for this player to execute a shot from this location, the ball has to be sent to a space in front of the opponent’s goal; however, this space is risky because it corresponds to (C) or (D).

Based on the ball-passing analysis discussed above, our framework also provides a new approach for field division based on the degrees of safety and sparsity. In particular, using the two axes, $z_{1}=0$ and $z_{2}=2$, the football field can be divided into the following four spaces: (A) safe dense space ($z_{1} > 0$ and $z_{2} < 2$), (B) safe sparse space ($z_{1} > 0$ and $z_{2} > 2$), (C) risky sparse space ($z_{1} < 0$ and $z_{2} > 2$), and (D) risky dense space ($z_{1} < 0$ and $z_{2} < 2$). We show a typical example of field division using our approach into spaces (A)–(D) in Fig. 5. From the figure, it can be confirmed that the dense spaces (A) and (D) defined as $z_{2} < 2$ are almost located within the formation, i.e., $\tilde{R} < 1$.

**Figure 6:**
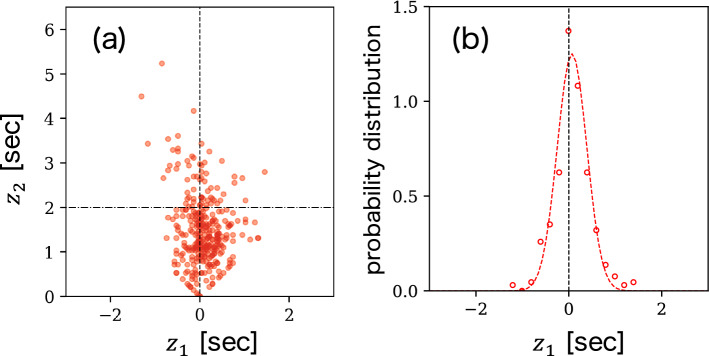
(**a**) Scatter plot of $z_{1}(\vec {x}_{\text{e}}, t_{\text{o}})$ and $z_{2}(\vec {x}_{\text{e}}, t_{\text{o}})$, and (**b**) probability distribution of $z_{1}(\vec {x}_{\text{e}}, t_{\text{o}})$, for successful passes just before the shots.

For example, in the case shown in Fig. 5, the player on the right side near the halfway line has the ball. In order for this player to execute a shot from this location, the ball has to be sent to a space in front of the opponent’s goal; however, this space is risky because it corresponds to (C) or (D). We show that passes just before shots have a tendency to become risky generally based on our framework. For passes just before shots, Fig. 6a,b present the scatter plots for $z_{1}(\vec {x}_{\text{e}}, t_{\text{o}})$ and $z_{2}(\vec {x}_{\text{e}}, t_{\text{o}})$, and the probability distribution of $z_{1}$ for successful passes, respectively. From Fig. 6b, we can observe that the peak value $z_{1} \simeq 0$ is smaller than that for the case of all passes ($z_{1} = 0.69$ in Fig. 3b). This observation indicates that passes just before a shot tend to become risky because the success probability of a pass is a monotonically increasing function of $z_{1}$, as shown in Fig. 3c. Here, Fig. 6 shows an average feature for various teams. Examining the same figure for an individual team will lead to team assessment in terms of the shot situation. For example, from the peak position in Fig. 6b for each team, we can evaluate how risky they are in shot situations.

As shown in Fig. 3c, the success probability $P(q=1|z_{1})$ of passes being well-fitted by the sigmoid function (5) could be a consequence of the normal distributions for $P(z_{1}|q=1)$ and $P(z_{1}|q=0)$ (see Fig. 3b). In particular, it is known that $P(q=1|z_{1})$ can be expressed as follows:

$$\begin{aligned} P(q=1|z_{1}) = \frac{1}{1 + \exp \left[ -\log \frac{P(z_{1}|q=1)P(q=1)}{P(z_{1}|q=0)P(q=0)}\right] }, \end{aligned}$$

which transforms to Eq. (5) when $P(z_{1}|q=1)$ and $P(z_{1}|q=0)$ follow a normal distribution with the same standard deviation^24^. In our case, the standard deviations obtained from fitting our data are $\sigma _{1} = 0.54$ and $\sigma _{0}=0.38$, and $\sigma _{1}\ne \sigma _{0}$. The deviation of the success probability curve shown in Fig. 3c from the sigmoid function curve where $z_{1} < 0$ can be attributed to this difference in standard deviation values. The sigmoid curve for $z_{1}$ can be applied to the player assessment in terms of difficulty of ball passings. For example, a player who completed many passes with $z_{1}<0$ can be evaluated as a good passer because such passes are difficult to complete by the sigmoid curve. In particular, $\sigma _{1}$ and $\sigma _{2}$ may be related to the passing abilities of players. Thus, new criteria for pass evaluation can be a typical practical application of our framework.

Finally, here, we present directions for future research related to the present study by comparing our framework with other relevant studies. First, Fernández and Bornn proposed a field weighting approach for space evaluation in football games^22^. In their method, a player’s influence on each location is defined by a multivariate normal distribution and is transformed into a single value to evaluate which team is dominant at a location. In contrast, our method evaluates a location in the field based on two variables, $z_{1}$ and $z_{2}$, which are defined based on the motion model. It is noteworthy that each of $z_{1}(\vec {x}, t)$ and $z_{2}(\vec {x}, t)$ has an explicit physical meaning, i.e., the degree of safety for a pass made to $\vec {x}$ at *t* and degree of sparsity of $\vec {x}$ at *t*, respectively. Several extensions to our proposed framework are required before it can be applied to real game analyses. For example, the information of the distances from the locations of the ball and goal should be incorporated into variables for field weighting as in the case of Fernández and Bornn’s approach, because the outcome of passes or shots typically depends on these distances. In regard to the motion of the ball, Spearman et al. proposed a model based on the equation of motion for the ball^20,21^. Our proposed framework could also be similarly extended to the ball, which would enable us to evaluate the accuracy of passes. Because our framework is independent of the definition of the motion model, we could employ any extended motion model based on the equations of motion or machine learning technique^18,19^ for calculating $z_{1}$ and $z_{2}$. A player-specific motion model might also allow us to assess the playing ability of players.

## Data Availability

The dataset (Player Tracking Data in J-League Matches) was obtained from DataStadium Inc., Japan; it is not publicly available, but it was provided to us based on an agreement with the company.
